# Clinical effects of COVID-19 vaccines on chronic spontaneous urticaria patients: a study on two Turkish centers

**DOI:** 10.3389/fmed.2023.1222126

**Published:** 2023-09-28

**Authors:** Demet Kartal, Shahzada Orujova, Ragıp Ertaş

**Affiliations:** ^1^Dermatology and Venereology Department, Erciyes University Medical School, Kayseri, Türkiye; ^2^Kayseri City Hospital Kayseri, Kayseri, Türkiye

**Keywords:** angioedema, chronic urticaria (CU), anaphylaxis, COVID-19 vaccination, vaccination

## Abstract

**Background:**

The relationship between CSU and vaccines is reported in the literature. The post-vaccine clinical effect of COVİD-19 vaccines on CSU patients is still unclear. we aimed to evaluate clinical effects of COVID-19 Vaccines on CSU patients.

**Materials and methods:**

CSU patients under treatment and follow-up in our outpatient clinic were evaluated retrospectively. To determine disease activity the urticaria activity score over seven days (UAS7) was used. The details of demographic information, CSU treatment, history of COVID-19 infection and exacerbation of urticaria during infection, COVID-19 vaccinations, type of vaccine, side effect reaction time after vaccination, hospital admission requirement were also assessed.

**Results:**

Total of 190 patients with a mean age of The mean age of patients was 41,7±14,0 years (range = 18–72) were enrooled. The patient sample consisted of 50 males and 140 females (male/female ratio 1:2.8). 181 (CoronaVac 77, Pfizer-BioNTech 104) patients were vaccinated. Urticarial exacerbation was occurred in 25 patients. The median UAS value was 5 before vaccination, it was 12 in patients with post-vaccine urticaria. Two patients had angioedema and one patient had anaphylaxis after vaccinated with Pfizer-BioNTech.

**Conclusion:**

COVID-19 vaccination in the CSU can be considered safe. Cases with urticarial exacerbation appear to be transient and can be managed by antihistamine and/or systemic steroid therapies. More extensive studies are needed for the relationship with CSU and post-coronavirus vaccination anaphylaxy.

## Introduction

Chronic spontaneous urticaria (CSU) is described as the occurrence of angioedema, wheals, or both for more than 6 weeks. It affects 1%–2% of the population (1). There are many factors that exacerbate CSU: medications, infections, hormonal changes, physical stimuli, and stress (2).

Many skin manifestations caused by Coronavirus disease 2019 (COVID-19) infection have been reported. The most common cutaneous patterns associated with COVID-19 include urticaria, maculopapular, vesicular, and livedoid lesions. Erythema multiforme (EM)-like lesions, multisystem inflammatory syndrome in adults (MIS-A), and multisystem inflammatory syndrome in children (MIS-C) have also been reported, albeit more rarely. Skin manifestations associated with COVID-19 was observed not only as a symptom of infection but also in relation to vaccines (3–5).

Various types of COVID-19 vaccines have been developed, including adenoviral vector vaccines, inactivated vaccines, mRNA vaccines, and subunit vaccines. Many systemic and local reactions have been observed in different platforms. The most common reported local reactions include swelling, erythema, and pain. Fever, fatigue, and headache are also commonly reported systemic reactions (3–5). The relationship between CSU and vaccines is reported in the literature (6), however, some publications have given different results about the effects of COVID-19 vaccines on patients with CSU (7–12).

In this study we aimed to evaluate the clinical effects of COVID-19 vaccines on patients with CSU.

## Materials and methods

CSU patients followed in our outpatient clinics (Erciyes University Medical Faculty Dermatology Department, Kayseri City Hospital Dermatology Department) were questioned retrospectively in their follow-up. Our study design was approved by the Erciyes University Review Board. To determine disease activity, the Urticaria Activity Score over 7 days (UAS7) was used during the CSU treatment and after vaccination. The details of demographic information, CSU treatment, comorbidities, history of COVID 19 infection and exacerbation of urticaria during infection, COVID-19 vaccinations, type of vaccine, reaction time after vaccination, and hospital admission requirement were also assessed.

Permission was asked of all participants to take part in the study and all provided their written and informed consent.

The data were evaluated in the statistical package program of IBM SPSS Statistics Standard Concurrent User V 26 (IBM Corp., Armonk, New York, United States). Descriptive statistics were given as number of units (*n*), percent (%), and mean ± standard deviation.

Chi-square exact test was used to compare groups with categorical variables. If the chi-square test results were found to be significant, subgroup analyses were performed with Bonferroni-corrected two-ratio Z-test. Pre-vaccine and post-vaccine UAS comparisons were made with the Wilcoxon test. The reaction rates to COVID-19 vaccines were compared with the One-sample Binomial test. A *p*-value of <0.05 was considered statistically significant.

## Results

A total of 190 patients with a previous diagnosis of CSU and/or angioedema were enrolled in this study from two centers. The patient sample consisted of 50 males and 140 females. The mean age of patients was 50.3 ± 11.6 years (range = 18–72; Table 1).

**Table 1:** Demographic characteristics of patients.

Characteristics	Number (%)
**Age**	
Mean	41.7 ± 14.0 years
Min, max	18–72 years
Female/Male	140/50
**Disease duration**	
Mean	85.5 ± 65.1 months
Min-max	24–276 months
**Pre vaccination urticaria treatment**	
Omalizumab	130 (%68)
Oma with AH	105 (%55)
Oma with AH when necessary	25 (%13)
AH	34 (%18)
Cyclosporine	13 (%7)
Without treatment	13 (%7)
Total vaccinated	168
Pfizer-BioNTech	101
CoronaVac	67
**Post vaccination reaction type**	
Urticaria	25
Angioedema	2
Anaphylaxis	1

AH, antihistamine; Oma, Omalizumab.

The duration of the disease is in the range of 24–276 months. The mean duration of the disease is 85.5 ± 65.1 months. Of the patients, 130 were under treatment with Omalizumab (68%), 105 received Antihistamines (AH) every day (55%), and 25 of them received AH when necessary (13%). In addition, 34 patients were treated with AH and 13 patients with Cyclosporine A (Table 1). While 177 (93%) patients were in follow-up with treatment, 13 (6%) patients were in follow-up without treatment with complete symptom control (0 score UAS 7). Of the patients, 85% had their disease well under control, with a UAS score < 6.

In total, 181 (CoronaVac 77, Pfizer-BioNTech 104) patients were vaccinated. Urticarial exacerbation occurred in 25 patients. All patients were treated with systemic steroids (0.5–1 mg/kg, 5–10 days) and AH (Table 2).

**Table 2:** Urticarial exacerbation rates after vaccination.

	Total	Urticarial flare up	*p*-value
	*N*	*N* (%)	
CoronaVac	77	6 (7.7)	<0.001^¥^
Pfizer-BioNTech	104	19 (18)	

*n*, patient number, %, percentage, ^¥^One sample binomial test.

**Figure 1:**
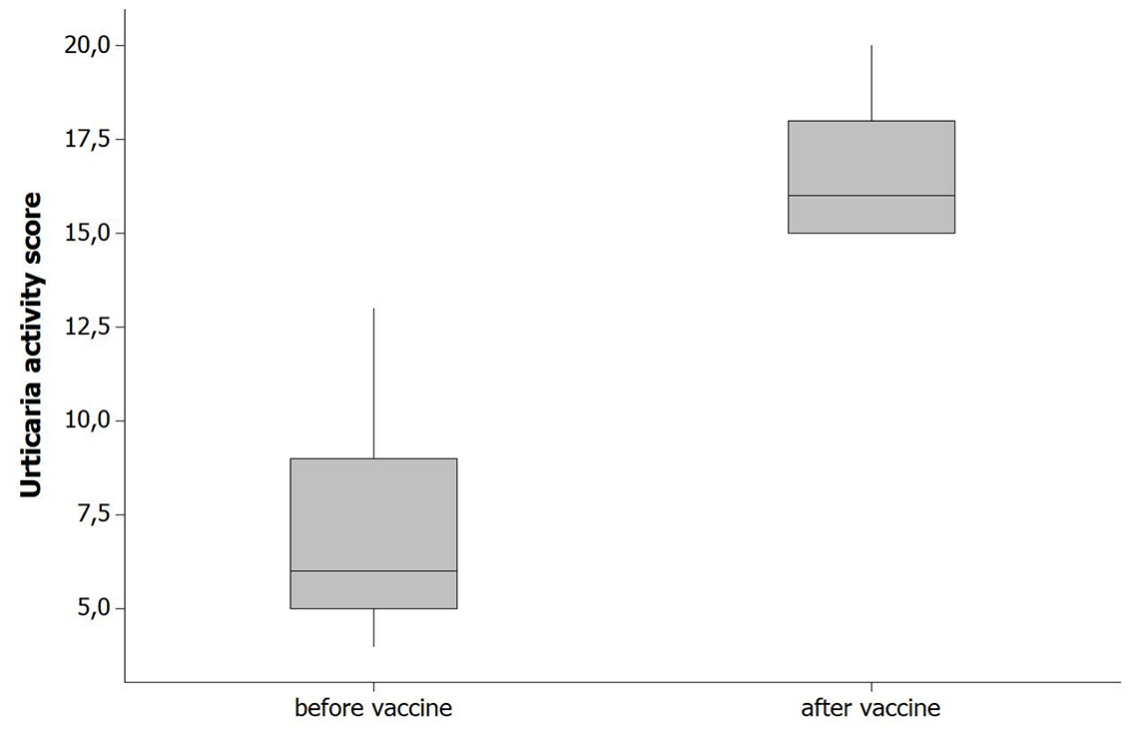
UAS values pre-vaccination and post-vaccination.

**Figure 2:**
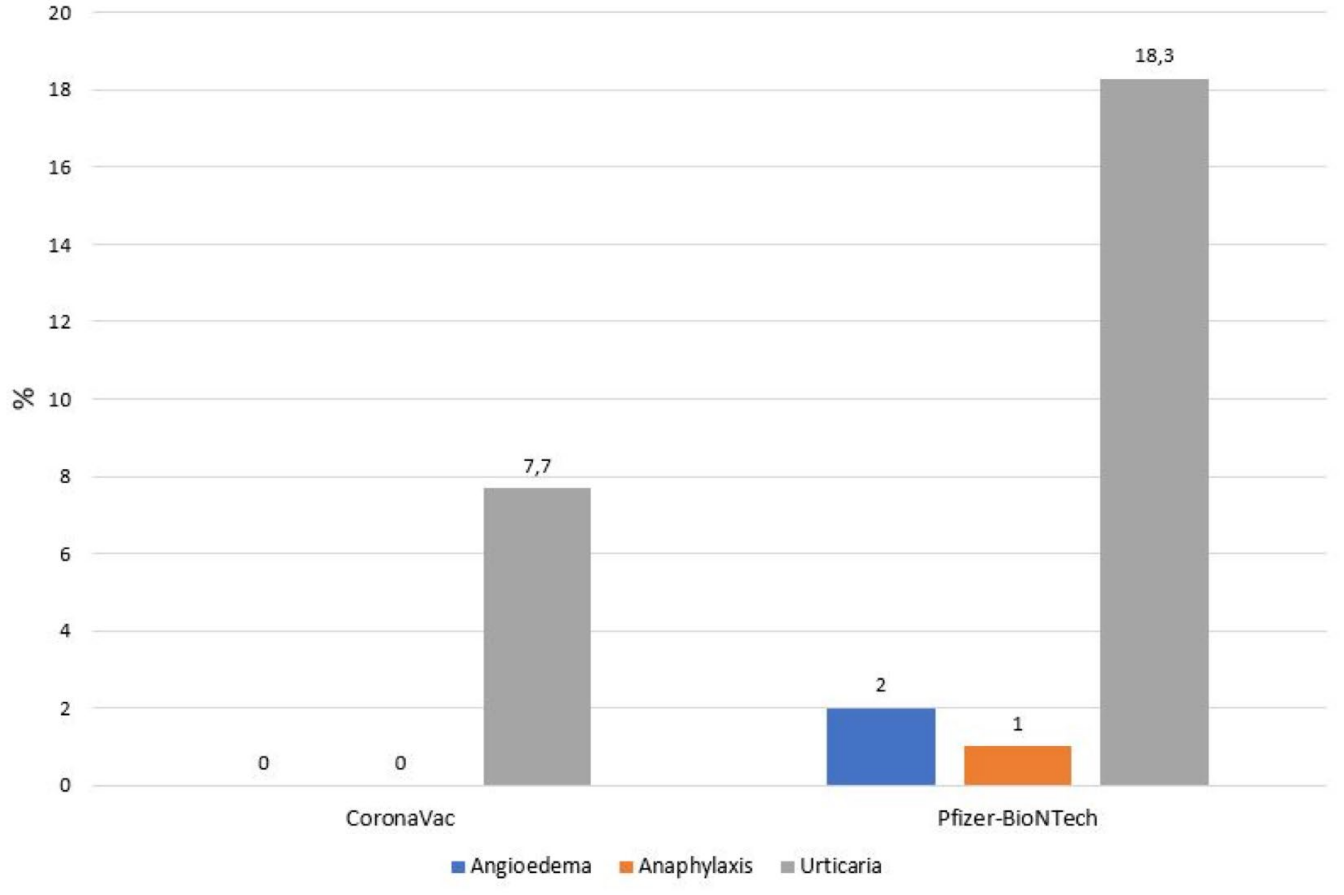
Post-vaccination reactions.

The reaction types that occurred after the vaccines and after which dose the reaction occurred are given in Table 3. The duration of reactions are given in Table 4. The comorbidity rates were 4.0% for autoimmune thyroid and 16.0% for asthma; these rates in general patients were 2.6% for autoimmune thyroid, 2.6% asthma, and 0.5% for allergic rhinitis. There was no statistical significance between the two groups (*p* > 0.05; Table 5). The median UAS value was 5 before vaccination, while it was 12 in patients with post-vaccine urticaria (Table 6 Figure 1) and was statistically significant (*p* < 0.01). Urticarial exacerbation occurred in six patients among those who received CoronaVac and in 19 patients who received Biontech. The occurrence of urticarial exacerbation after Pfizer-BioNTech vaccine was higher than the occurrence of urticarial exacerbation after CoronaVac, and this was statistically significant (*p* < 0.001; Table 2; Figure 2). None of the patients took any other medication before vaccination. Of the patients with a post-vaccine reaction, 10 continued with routine vaccination, while three patients proceeded with an alternative vaccine. No post-vaccine reaction was observed in any of these patients. The post-vaccine reaction types and for which dose the reaction occurred are given in Table 3.

**Table 3:** Vaccine doses and types of reactions.

Pfizer-BioNTech	Patient number (*n*)
**Reaction dosage**	
1.Dosage	18
2.Dosage	3
3.Dosage	1
**Reaction type**	
Urticaria	19
Angioedema	2
Anaphylaxy	1
**CoronaVac**	
**Reaction dosage**	
1.Dosage	3
2.Dosage	3
**Reaction type**	
Urticaria	6
Angioedema	0
Anaphylaxy	0

**Table 4:** Percentages in terms of reaction times in patients with post-vaccine urticarial exacerbation.

(*n* = 25)	*n* (%)
0–30 min	13 (52.0)
30 min–24 h	8 (32.0)
24 h–1 week	4 (16.0)

*n*, patient number; %, percentage.

**Table 5:** Patients’ comorbidities.

		Otoimmun thyroiditis	Astma	Allerjik rhinitis
	*N*	*n* (%)	*n* (%)	*n* (%)
All patients	190	5 (2.6)	6 (3.1)	1 (0.5)
Patients with reaction after vaccination	25	1 (4.0)	2(8.0)	0 (0.0)
		*p* = 0.421^†^	*p* = 0.52^†^	

*N*, total patient number; *n*, patient number; %, percentage, ^†^Kikare exact test.

**Table 6:** UAS values pre vaccination and post vaccination in patients with flare up after vaccination.

	Values		*p*-value
	Pre vaccination	Post vaccination	
UAS	6.0 (3.5)	16.0 (3.0)	<0.001^&^

Data are given as median (interquartile range) values. ^&^Wilcoxon testi.

Two patients had angioedema with Pfizer-BioNTech; they had urticaria previously and were under control with Omalizumab. Both patients were treated in the emergency departments of the hospitals where they were vaccinated. One patient had anaphylaxis after being vaccinated with Pfizer-BioNTech. She had been treated for 4 years with Omalizumab. This patient had exacerbations while receiving Omalizumab therapy. The median time until onset of exacerbation was 5 min. She developed flushing, urticaria on her whole body, and syncope. She was hospitalized and was treated in the intensive care unit for 24 h. She was discharged in good condition 1 day later.

## Discussion

Quantifying, identifying, and weighing proven and possible safety risks against potential advantages are important aspects of developing any vaccine (13). It is essential to take the necessary time to make medical and regulatory decisions while focusing on profit and risk estimates, as well as identifying the stakes and possible checkpoints during vaccine development periods. In addition to not interrupting the vaccination course, the detection of diseases in the risk group in terms of exacerbation is also important. In this study, 190 patients diagnosed with CSU were evaluated in terms of possible urticarial activation after vaccination. The female/male ratio was 140/50. We believe significantly more women participated in this study because the physician who monitored the patients was also a woman.

In Turkey, the vaccinations against severe acute respiratory syndrome coronavirus 2 (SARS-CoV-2) started with an inactivated virus vaccine CoronaVac developed by Sinovac Life Sciences in China. Due to the limited numbers of the first imported vaccine and the active service of health workers, vaccination priority was given to healthcare workers followed by the elderly and other professions. A few months later, with the approval of the other imported vaccine Pfizer-BioNTech (BNT162b2), the vaccinations continued with CoronaVac vaccine and Pfizer-BioNTech vaccine. Many reports on the effectiveness of vaccines, their use in special patient groups, side effects, and other features have been published in the literature. Dermatological side effects of vaccines and exacerbations of dermatological diseases with which they are associated were also studied (3–5). In our study, a total of 181(104 Pfizer-BioNTech, 77 CoronaVac) patients were vaccinated. 67.7% (6/77) of patients had urticarial exacerbation after CoronaVac. In a study, authors mentioned that atopic dermatitis and thyroid diseases are risk factors for immediate hypersensitivity reactions (IHSR) after COVID-19 vaccinations. Our results do not support this relationship (11). All patients with urticarial exacerbation after vaccinations were treated with systemic steroids (0.5 mg–1 mg/kg, 5–10 days) and AH.

Angioedema did not occur in any patient after vaccination. There is little data in the literature reporting the occurrence of urticaria after CoronaVac vaccine (12–14). In a tertiary university hospital in Turkey, health workers showed a hypersensitivity reaction after CoronaVac vaccine (12). Authors reported one angioedema, one anaphylaxis, two urticaria, and three hypersensitivity symptoms in 5,558 doses.

In another study, exacerbation of urticaria after inactivated vaccinations was reported. Authors reported that 11.1% (5/45) of patients had urticarial exacerbation after the first dose and 8.9% (4/45) after the second dose of inactivated vaccines (13).

There are publications in the literature reporting the development of urticaria after the Pfizer-BioNTech vaccine (6, 9). However, there is little literature on urticaria exacerbation in CSU patients after the Pfizer-BioNTech vaccine (15, 16).

In a study with 90 participants, authors reported urticarial exacerbations after the Pfizer-BioNTech vaccine. In this study,14 (15.5%) patients experienced exacerbations in their urticarial activity after the first or repeated doses of Pfizer-BioNTech vaccinations. After the first dose, 10 (11.1%) out of 90 patients had an urticarial reactivation while five (7.9%) of 63 patients experienced reactivation after the second dose. As a result, the Pfizer-BioNTech vaccine triggered CSU reactivity in about 15.5% of CSU patients in the short term following the vaccination. In addition, 32 new-onset and 27 relapsed CSU patients within 3 months after Pfizer-BioNTech vaccination were retrospectively assessed (15).

In a study from Italy, Chronic Spontaneous/Inducible urticaria (CSU/CIU) exacerbation was reported with the Comirnaty/Moderna vaccine, which is another mRNA vaccine. Here, 160 CSU/CIU patients who were under control with Omalizumab/antihistamine therapy received two doses of Comirnaty/Moderna vaccines. After vaccine administration, 13 patients (8.12%) experienced exacerbation of CSU symptomatology (16). In our study, 18% (19/104) of patients experienced CSU exacerbation. Two patients had angioedema in the Pfizer-BioNTech vaccine group. The position paper on Allergies and COVID-19 vaccines published by the European Network of Drug Allergies (ENDA)/European Academy of Allergy & Clinical Immunology (EAACI) recommends routine vaccination to patients with post-vaccine urticaria and angioedema (17). In our study, 10 of the patients with post-vaccine urticaria and angioedema continued with routine vaccination, while three patients requested alternative vaccination. No post-vaccine reaction was observed in any of them. All patients were treated with systemic steroids (0.5–1 mg/kg) and AH. In a report, four patients with post-(mRNA)vaccination urticaria were treated with systemic steroids (8).

We know that many factors are triggers for urticaria (2). The fact that the patients did not take any medication before the vaccine reduces the possibility of another trigger being responsible.

In our study, anaphylaxis was observed in only one patient with the Pfizer-BioNTech vaccine. A strong predominance with both mRNA vaccines in women was observed (17) and our patient was also a woman. She was on Omalizumab therapy with CSU, and she had mild symptoms under treatment. Within 5 min of vaccine administration, she developed flushing, urticaria on her whole body, and syncope. She was monitored in an intensive care unit. According to the ENDA/ EAACI position paper on Allergies and COVID-19 vaccines, it is recommended to undergo a prick test for patients who have a history of an immediate (<2 h) or severe allergic reaction (anaphylaxis) to a vaccine containing (PEG), polysorbate, or-polyoxyl 35 castor oil (17). After recovery, our patient was referred to the allergology department for evaluation with a Prick test [vaccine, Polyethylene glycols (PEG)] but she refused to go to the allergology and stated that she did not want to be vaccinated again.

In a systematic review, authors performed a Vaccine Adverse Event Reporting System (VAERS) database search, looking for events reported between December 2020 and May 2022. They estimated the publicly reported rates and compared them with the currently published rates of anaphylaxis for the three authorized vaccines in the United States (Moderna, Pfizer-BioNTech, and Janssen). According to reports, 2,320 anaphylactic events were reported in the database for a total of 586 million doses administered. The anaphylaxis rate in 4/million doses for all COVID-19 vaccines was estimated. The rate for the Pfizer-BioNTech vaccine was 3.7/million. Overall, the highest number of anaphylactic events was reported for Pfizer-BioNTech (55%), followed by Moderna (38%) and Janssen (7%) (18).

In conclusion, COVID-19 vaccination for patients with CSU can be considered safe. Cases with urticarial exacerbation appear to be transient and can be managed by antihistamine and/or systemic steroid therapies. More extensive studies are needed to establish the relationship between CSU and post-coronavirus vaccination anaphylaxis.

## Data availability statement

The raw data supporting the conclusions of this article will be made available by the authors, without undue reservation.

## Ethics statement

The studies involving humans were approved by Erciyes University Ethical Committee. The studies were conducted in accordance with the local legislation and institutional requirements. Written informed consent for participation was not required from the participants or the participants’ legal guardians/next of kin in accordance with the national legislation and institutional requirements.

## Author contributions

DK: conceptualization, project administration, writing—original draft preparation, writing—review and editing, and visualization. DK and SO: methodology. SO: software, formal analysis, investigation, and resources. DK and RE: validation. All authors contributed to the article and approved the submitted version.
